# Advanced treatment strategies for high-altitude pulmonary hypertension employing natural medicines: A review

**DOI:** 10.1016/j.jpha.2024.101129

**Published:** 2024-10-25

**Authors:** Zahra Batool, Mohammad Amjad Kamal, Bairong Shen

**Affiliations:** a Center of High Altitude Medicine, West China Hospital, Sichuan University, Chengdu, 610041, China; b Department of Pharmacy, Faculty of Health and Life Sciences, Daffodil International University, Dhaka, 1216, Bangladesh; c Centre for Global Health Research, Saveetha Medical College and Hospital, Chennai, Tamil Nadu, 600001, India

**Keywords:** High-altitude illness, High-altitude pulmonary hypertension, Chinese medicine, Natural remedies, Hypoxia, Pulmonary artery pressure

## Abstract

High-altitude pulmonary hypertension (HAPH) occurs when blood pressure in the pulmonary arteries rises due to exposure to high altitudes above 2,500 m. At these elevations, reduced atmospheric pressure leads to lower oxygen levels, triggering a series of physiological responses, including pulmonary artery constriction, which elevates blood pressure. This review explored the complex pathophysiological mechanisms of HAPH and reviewed current pharmaceutical interventions for its management. Meanwhile, this review particularly emphasized on the emerging research concerning Chinese medicinal plants as potential treatments for HAPH. Traditional Chinese medicines are rich in diverse natural ingredients that show significant promise in alleviating HAPH symptoms. We reviewed both *in vitro* and *in vivo* studies to assess the efficacy, safety, and mechanisms of these natural medicines, along with their potential adverse effects. Additionally, this review highlighted new alternative natural remedies, underscoring the need for ongoing research to expand available treatment options for HAPH.

## Highlights

• The pathophysiology of HAPH has been reviewed.
• Pharmaceutical drugs for HAPH treatment pose adverse side effects.
• Chinese natural medicinal plants hold promise for alleviating HAPH symptoms.
• Evaluating the safety and efficacy of natural medicines is challenging.

## Graphical abstract

**Figure:**
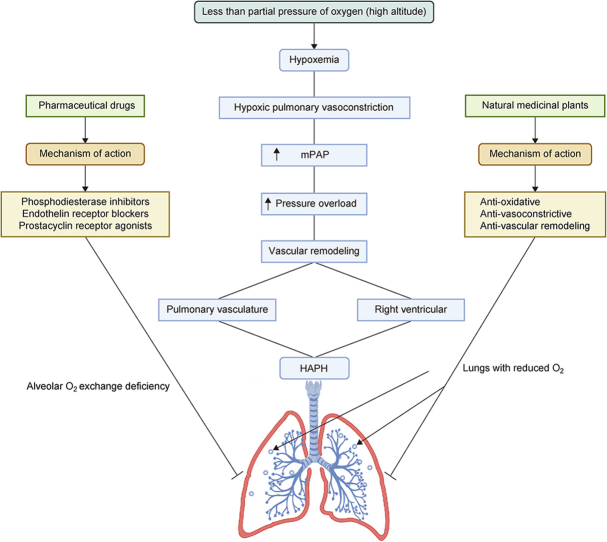

## 1 Introduction

High-altitude illness (HAI) encompasses a range of maladaptive syndromes, such as high-altitude mountain sickness (AMS), high-altitude pulmonary hypertension (HAPH), and high-altitude pulmonary edema (HAPE), manifesting at altitudes above 2,500 m [1,2]. Certain factors, such as genetics, altitude, physical exertion, and individual physiology, are the main contributors of the individuals susceptibility to HAI [[3], [4], [5]]. AMS is the mildest and most common HAI, characterized by symptoms such as headache, nausea, and dizziness that arise within hours and up to a day of rapid ascent, and generally resolves with acclimatization or gradual ascent [6]. However, HAPH is a chronic condition, developing over weeks to months of high-altitude exposure, causing sustained elevated blood pressure in the pulmonary arteries that produces symptoms such as persistent shortness of breath and fatigue [7]. In contrast, HAPE is an acute and life-threatening condition that arises within days of ascent, marked by fluid accumulation in the lungs, severe respiratory distress, and a productive cough with pink frothy sputum [8]. Hence, HAI manifests as mild AMS, chronic HAPH, and severe, life-threatening HAPE, each characterized by distinct symptoms and severity levels.

An elevated mean pulmonary artery pressure (mPAP) ≥ 25 mmHg at rest, measured by right heart catheterization, is considered diagnostic for pulmonary hypertension (PH) [9]. However, a precise elevated mPAP of 30 mmHg combined with a pulmonary artery wedge pressure of 15 mmHg and pulmonary vascular resistance (PVR) ≥3 Wood units (WU) represents HAPH [10,11]. In the latest PH classification guidelines, HAPH was categorized as category III HAI [10,12]. Clinical presentations commonly associated with HAPH pathology include headache and dyspnea accompanied by polycythemia and hypoxemia [[13], [14], [15]]. However, in advanced stages, potential risk factors such as congestive heart failure may emerge [[13], [14], [15]].

A number of pharmaceutical drugs, such as bosentan, ambrisentan, and macitentan (endothelin (ET) receptor antagonists (ERAs)) [[16], [17], [18]]; sildenafil (phosphodiesterase-5 inhibitor) [19]; and selexipag (prostacyclin receptor agonist) [20], can be used to treat HAPH. (Table 1 [[16], [17], [18], [19], [20]]). However, these pharmaceutical options cannot be used without limitations due to adverse side effects, and therefore should be used with caution [21].

**Table 1:** Pharmacological drugs for high-altitude pulmonary hypertension (HAPH) treatment with their mechanism of action and side effects.

Drugs category	Drug name	Mechanism of action	Side effects	Refs.
ET receptor blockers	Bosentan	It functions as a selective, competitive antagonist for both ET_A_ and ET_B_ receptors	Edema, anemia and transaminase elevation., headache flushing, syncope, hepatic dysfunction, impared renal tubular function, palpitation, fatigue, arthralgia, dyspepsia, and epistaxis	[16]
	Ambrisentan	It specifically targets ET_A_ receptors, preventing vasoconstriction mediated by these receptors on VSMCs	Headache, swollen feet and ankles, edema, hepatotoxicity, teratogenicity, and anemia	[17]
	Macitentan	It functions as a dual ERA, thereby inhibiting ET-1 binding to both ET_A_ and ET_B_ receptors	Headache, anaemia and bronchitis, and hepatic dysfunction	[18]
Phosphodiesterase inhibitors	Sildenafil citrate	It acts by blocking NO breakdown, promote pulmonary vasodilation, decreasing mPAP and PVR	Ventricular arrhythmia, gastrointestinal upset, muscle aches and joint pains, headache, and flushing	[19]
Prostacyclin receptor agonists	Selexipag	It acts as prostacyclin IP receptor agonist by providing its vasodilatory effects in PH	Headache, diarrhea, nausea, and jaw pain	[20]

ET: endothelin; ET_A_: endothelial ET receptor A; VSMCs: vascular smooth muscle cells; ERAs: ET receptor antagonists; NO: nitric oxide; mPAP: mean pulmonary artery pressure; PVR: pulmonary vascular resistance; IP: Inositol 1,4,5-trisphosphate; PH: pulmonary hypertension.

Natural medicines exhibit diverse pharmacological properties [22] and the abundance of natural medicinal resources at high-altitude regions render them popular among local population. Approximately 80% of the global population relies on medicinal plants, which served as vital resources for addressing healthcare needs [23]. While numerous medicinal plants are considered promising options for preventing and treating HAPH [22], their appeal lies in their potential to offer effective remedies with minimal or no side effects. With the increasing interest in medicinal plants and the limitations of current anti-HAPH therapies, there is a growing need to explore the mechanisms, effectiveness, and safety profiles of these herbal medicines [22,23].

This paper sets out to explore the pathophysiology of HAPH, followed by reviewing pharmaceutical drugs aimed at its mitigation. However, special attention is dedicated to the burgeoning research surrounding Chinese medicinal plants as potential remedies for alleviating HAPH symptoms by reviewing different *in vitro* and *in vivo* model studies along with their effects on activities and mechanisms of natural medicines. Additionally, this discussion delved into safety considerations associated with the utilization of natural medicines, as well as the emergence of new alternative natural candidates within this realm.

## 2 Pathophysiology of HAPH

HAPH pathology is characterized by having a precise elevation cut-off point of mPAP of 30 mmHg at high altitude in individuals without any previous symptoms of pulmonary or heart disease [10,24]. Individuals exposed to high altitudes develop hypoxemia due to the low ambient partial pressure of oxygen. This hypoxic stress response is characterized by an increased cardiac output, hypoxic ventilatory response, and increased hemoglobin concentration in response to the diminished oxygen diffusion driving force between lungs and tissue capillaries [25]. Moreover, changes in mitochondrial and capillary densities along with certain alterations in metabolic efficiency become evident at the cellular level [26].

Hypoxia-inducible factors (HIFs) being comprised of α and β subunits are heterodimeric transcription factors. They play a key role in triggering the body's response towards low oxygen levels [27,28]. Both subunits as member of PAS family of basic helix-loop-helix transcription factors are continuously transcribed and translated. In oxygen-rich environments, prolyl hydroxylases (PHDs) caused the hydroxylation of HIF subunits at proline residues. This modification lead them to be targeted for degradation by an E3 ubiquitin ligase through the von Hippel-Lindau protein complex [29]. Meanwhile, activity of PHDs depends on the level of oxygen. Under hypoxic conditions, PHD activity decreases, allowing HIF proteins to remain stable and activate the transcription of numerous genes [10,29]. Consistently, both HIF subunits are highly expressed in pulmonary endothelial cells, where they play a critical role in the expression of mitogenic factors, vasoconstrictors, and their receptors. These include placental growth factor (PGF), vascular endothelial growth factor (VEGF), angiopoietin-1 receptor (ANGPT1), ANGPT2, platelet-derived growth factor (PDGF), and serotonin [30,31]. Moreover, pulmonary vascular endothelial cells (PVECs) altered and increased phosphorylated signal transducer and activator of transcription 3 (STAT3) expression and its downstream pro-survival target, Mcl-1, during PH [32]. In contrast, hypoxic pulmonary artery smooth muscle cells (PASMCs) exhibited elevated expression of VEGF compared with endothelial cells. Moreover, PASMCs demonstrated reduced expression of ANGPT2, while the expression levels of PDGF and ANGPT1 remained unchanged [30]. Hence, indicating that HIF-targeted genes exhibited varying expression patterns across different cell types. In addition, chronic hypoxia induces VEGF expression, a potent inducer of angiogenesis, in a manner dependent on HIF-1α or HIF-2α [33]. The regulation of mitogens by HIF involves various growth factors (fibroblast growth factor 2 (FGF-2), PDGF, epidermal growth factor (EGF), and thrombin), vascular substances (ET-1), and cytokines. These factors induced HIF-1 target gene's expression via phosphatidylinositol 3-kinase or mitogen-activated protein kinase (MAPK) signaling pathways, creating a positive feedback loop that amplified HIF activity during hypoxia [33].

Hypoxia-induced HAPH might also be caused by increased reactive oxygen species (ROS) due to decreased oxygen levels in the lungs, leading to acute or chronic hypoxia [34]. ROS sources include complex III in the mitochondrial electron transport chain, cytochromes, and plasma membrane-bound nicotinamide adenine dinucleotide phosphate (NADPH) oxidases. This phenomenon underscored the complex interplay of physiological factors contributing to HAPH and emphasized the role of oxidative stress (OS) in its pathogenesis [35]. Initially, OS responses result in elevated ROS levels and malondialdehyde (MDA), an oxidative damage marker. Concurrently, levels of antioxidative markers such as superoxide dismutase (SOD) and glutathione (GSH) are diminished [35]. This decrease in antioxidative markers might be due to the interaction between hemoglobin and SOD, causing an increased production of ROS. Consequently, this cascade of events can culminate in inflammation and endothelial cell damage during HAPH [36].

Moreover, pulmonary circulatory response to alveolar hypoxia is the redistribution of blood flow within the parenchyma of the lungs to regions that are well oxygenated [4]. This phenomenon, known as hypoxic pulmonary vasoconstriction (HPV), was first observed in cats by Penaloza and Arias-Setlla [37]. Subsequently, in areas of focal hypoxia, HPV optimized ventilation perfusion to improve pulmonary gas exchange. However, HPV induced systemic pulmonary vasoconstriction, when oxygen availability declines, leading towards rapid and reversible increase in mPAP. Evidencing that, PH occurs during chronic or intermittent hypobaric hypoxia mainly following the disruption of three critical signaling pathways: nitric oxide (NO), prostacyclin-thromboxane A_2_ (PTA_2_), and ET-1 pathways [[38], [39], [40]].

### 2.1 NO pathway

NO served as a vital molecule, being produced by endothelial NO synthase (eNOS) in the endothelial cells [38,41]. Its synthesis happens via oxidation of l-arginine to l-citrulline, involving oxygen, NADPH, and various essential cofactors. Following its synthesis, NO diffuses to the adjacent pulmonary vascular smooth muscle cells (PVSMCs) [42], where it binded to soluble guanylate cyclase (sGC), initiating a cascade, converting guanosine triphosphate (GTP) into cyclic guanosine monophosphate (cGMP). This interaction is pivotal in regulating various physiological processes, particularly those modulating vascular function, in which the signaling pathway facilitated vasodilation and maintained blood pressure. This also activated downstream cGMP-dependent protein kinase (PKG), which eventually resulted in pulmonary vasodilation. In addition, NO exerted inhibitory effects on PVSMC proliferation, platelet aggregation, and thrombosis, collectively contributing to the maintenance of a healthy pulmonary vascular system [43].

In HAPH, there is a reduction in the bioavailability of NO, leading to vasoconstriction and an increase in inflammation, smooth muscle cells (SMCs) proliferation, and potentially thrombosis. Firstly, a decreased expression of endothelial eNOS was found to be associated with these pathological changes in HAPH [44]. However, further studies revealed similar results for sustained eNOS activation in both mice models and human studies [45]. A possible explanation is the influence of ROS, with a specific focus on tetrahydrobiopterin (BH_4_), in the enzymatic decoupling of eNOS [46]. BH_4_, as a eNOS cofactor, promoted the eNOS dimer formation as well as stabilized enzyme in its active dimeric form, which optimally oxidized l-arginine into NO. However, a decreased bioavailability of BH_4_ leads towards eNOS decoupling, producing a dysfunctional state [47]. When eNOS dimers destabilized, the resulting eNOS monomers produced ROS instead of NO, leading to hypertension.

Interestingly, the bioavailability of BH_4_ depends on the balance between its *de novo* synthesis by GTP cyclohydrolase-1 (the first and rate-limiting step in the biosynthesis of BH_4_ from GTP), its loss through oxidation into dihydrobiopterin (BH_2_), and BH_2_ recycling back into BH_4_ by dihydrofolate reductase (DHFR) [48]. Even in the absence of BH_4_ deficiency, increased levels of BH_2_ can compete with BH_4_ for binding to eNOS, resulting in the decoupling of eNOS. In this context, both the stoichiometry of eNOS/BH_4_ and the redox status of biopterin have been identified as key determinants of eNOS decoupling, rather than the absolute concentration of BH_4_ [49]. Therefore, deficiency in BH_4_ lead to increased pulmonary vascular tone by reducing eNOS activity and NO bioactivity, which in turn promoted vascular remodeling, right ventricular hypertrophy, and hypertension [48,49].

### 2.2 PTA_2_ pathway

Prostaglandin I_2_ (PGI_2_) or prostacyclin is synthesized from arachidonic acid within endothelial cells through the action of cyclooxygenase and prostacyclin synthase enzymes. Once formed, PGI_2_ binded to the specific I-prostaglandin (IP) receptors on SMCs, initiating a cascade of events. This binding activated adenylyl cyclase, an enzyme responsible for converting adenosine triphosphate (ATP) into cyclic adenosine monophosphate (cAMP). The accumulation of cAMP resulted in smooth muscle relaxation, which facilitated vasodilation. In addition, PGI_2_ reduced SMC proliferation and aggregation of platelets, while exerting anti-thrombotic and anti-inflammatory effects [39].

During HAPH, there is a significant shift in the metabolic pathway towards the production of thromboxane A_2_ as an alternative product. This shift attributed to the altered pharmacological behavior of prostacyclin under hypoxic conditions [50]. Normally, prostacyclin preferentially bind to its specific IP receptors, producing vasodilation and inhibiting platelet aggregation. However, during hypoxia, the interaction of prostacyclin with its receptors becomes dysregulated. Prostacyclin begins activating other prostanoid receptors, such as thromboxane receptors, particularly when IP receptors are limited [51,52]. This aberrant activation caused an overproduction of thromboxane A_2_, which contributed to a pathological imbalance between thromboxane and prostacyclin. Under normal conditions, prostacyclin and thromboxane have opposing effects: prostacyclin promoted vasodilation and inhibited platelet aggregation; whereas thromboxane A_2_ promoted vasoconstriction and platelet aggregation. When prostacyclin started to mimic the effects of thromboxane through aberrant activation, the prostacyclin–thromboxane balance got disrupted, leading to increased platelet aggregation, enhanced pulmonary vasoconstriction, and excessive proliferation of pulmonary vascular endothelial and SMCs [53].

The critical roles of prostacyclin were further elucidated through studies using mice models [54]. For example, genetically engineered mice lacking the IP receptor exhibited severe manifestations of hypertension, including pronounced vascular remodeling in response to chronic hypoxia [54,55]. This underscored the importance of the prostacyclin-IP receptor pathway for maintaining vascular homeostasis and mitigating the effects of hypoxia. Human patients with HAPH are also similarly compromised. They showed a notable reduction in prostacyclin synthesis with decreased expression of both the IP receptor and prostacyclin synthase [51,55]. This reduction exacerbated the imbalance between thromboxane and prostacyclin, contributing to the pathophysiology of HAPH [52]. Hence, the treatment requirs critical therapeutic strategies to restore the normal function of prostacyclin and its receptor to counteract the adverse effects of HAPH.

### 2.3 ET-1 pathway

ET-1, as a peptide, is well known for its potent vasoconstrictive effects [56,57]. It is produced from a precursor peptide, big ET-1, via ET converting enzymes present on the endothelial cell membrane. After being secreted, ET-1 interacted with two G protein-coupled receptors, ET_A_ and ET_B_. ET_A_ is found on vascular SMCs, where it promoted hypertrophy, vasoconstriction, cellular proliferation, and fibrosis upon activation [40]. ET_B_ is distributed on both vascular SMCs and endothelial cells. ET_B_ receptor activation on SMCs induces vasoconstriction, which triggered NO and prostacyclin production in endothelial cells, leading to vasodilation and anti-proliferative effects [40].

In HAPH pathology, there is an upregulation of both contractile ET_A_ and ET_B_ receptors in SMCs, and downregulation of diastolic ET_B_ receptors in the endothelium [58]. In addition, patients with HAPH exhibited elevated concentrations of ET-1 in plasma and PVECs [59,60]. This imbalance, marked by heightened ET_A_-mediated vasoconstriction and reduced ET_B_-mediated vasodilation, leads to an increased vascular tone and the proliferation of PASMCs [60]. Hence, an increased activation of the ET-1 signaling pathway, particularly through the stimulation of ET_A_ receptors, played a crucial role in the progression of PH. Moreover, ET_A_ receptors, when activated by ET-1, promoted vasoconstriction and proliferation of SMCs, leading to increased PVR. ET-1 activation of ET_B_ receptors in pulmonary artery endothelial cells (PAECs) also contributed to the HAPH pathology. This activation stimulated the expression of Rho-kinase (ROCK), an enzyme that promoted contraction and proliferation of vascular SMCs (VSMCs). At the same time, there is suppression of peroxisome proliferator-activated receptor-γ (PPAR-γ), a nuclear receptor that typically inhibited inflammation and SMCs proliferation. Hence, effect of increased ROCK activity and decreased PPAR-γ expression might increased vasoconstriction and vascular remodeling [61]. Given these mechanisms, both ET_A_ and ET_B_ receptors activation are implicated in the pathogenesis of HAPH. ET_A_ receptor activation primarily drives vasoconstriction and SMCs proliferation, and ET_B_ receptor activation on endothelial cells contributed to these processes through different molecular pathways, exacerbating the disease [62].

Pathological changes to the ET-1 signaling pathway can be mitigated using ERAs. These antagonists can selectively target ET_A_ receptors or act on both ET_A_ and ET_B_ receptors [62]. By blocking these receptors, ERAs reduced vasoconstriction and SMCs proliferation, thereby alleviating the symptoms of PH. Notably, a combined blockage by ET_A_ and ET_B_ receptors might be more effective than solely inhibiting ET_A_ receptors. This dual inhibition approach more comprehensively addressed the complex mechanisms involved in HAPH, including the ET_B_-mediated modulation of ROCK and PPAR-γ expression in PAECs [62]. By targeting both receptors, it might be possible to achieve significant reduction in vascular tone and remodeling, leading towards better clinical outcomes for patients with HAPH.

In summary, the development of HAPH can be attributed to several key factors. These factors included decreased production of PGI_2_ due to dysregulation of cyclooxygenase-2, leading to impaired vasodilation through dysfunction of eNOS [61,63]. In addition, an upregulation of ET-1 signaling contributed to enhanced vasoconstriction and mitotic effects, further exacerbating HAPH [61,63]. The enhanced understanding of these three primary pathways has greatly propelled the development of targeted drug therapies for HAPH. Consequently, there has been a rapid expansion in both the quantity and efficacy of available treatment options.

## 3 Pharmaceutical drugs in the management of HAPH

**Fig. 1:**
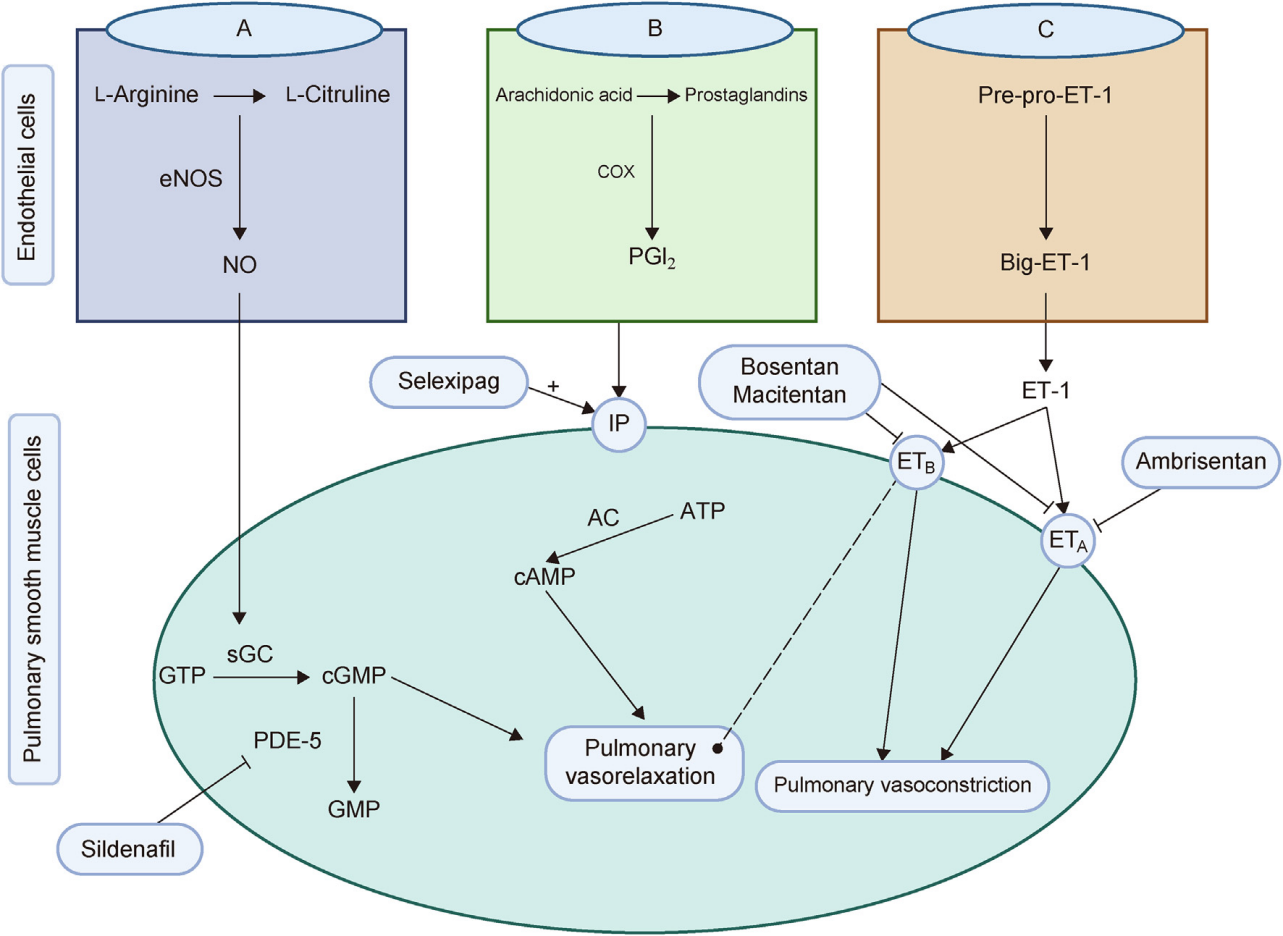
The pharmacological treatment of high-altitude pulmonary hypertension (HAPH) targets specific abnormal pathways, with contemporary drugs acting through various mechanisms: (A) nitric oxide (NO) pathway, (B) prostacyclin pathway, and (C) endothelin (ET) pathway. The dashed line originating from endothelial ET receptor B (ET_B_) signifies the results of endothelial ET_B_ activation, leading to the production of NO and prostaglandin I2 (PGI_2_). eNOS: endothelial NO synthase; COX: cyclooxygenase; IP: PGI_2_ receptor; GTP: guanosine triphosphate; sGC: soluble guanylate cyclase; cGMP: cyclic guanylate monophosphate (GMP); PDE-5: phosphodiesterase-5; ATP: adenosine triphosphate; AC: adenylyl cyclase; cAMP: cyclic adenosine monophosphate (AMP). Figure created by BioRender.com.

Managing HAPH can be challenging due to limited data availability; however, treatments used for other types of PH have shown promise for improving symptoms and mPAP in HAPH pathology. The main pharmacological treatments for HAPH include phosphodiesterase inhibitors, ET receptor blockers, and prostacyclin receptor agonists (Fig. 1).

### 3.1 Phosphodiesterase inhibitors

Sildenafil citrate, a phosphodiesterase type 5 (PDE5) inhibitor, induced SMCs relaxation in the pulmonary vasculature and in the corpus cavernosum [19,64]. By blocking the breakdown of NO, sildenafil promoted pulmonary vasodilation in patients with HAPH. Subsequent, previous studies [19,64,65] demonstrated that sildenafil decreased mPAP and PVR in HAPH pathology.

### 3.2 ET receptor blockers

Bosentan, ambrisentan, and macitentan are categorized as ET receptor blockers [66,67]. Each has its own unique pharmacological profile and mechanism of action.

Bosentan is a non-peptide pyrimidine derivative, acting as a competitive and specific antagonist for both ET_A_ and ET_B_ receptors. It was the pioneer ERA drug approved for PH treatment, specifically in patients classified as World Health Organization (WHO) functional classes III and IV. By antagonizing both ET_A_ and ET_B_ receptors, bosentan counteracted the vasoconstrictive effects of ET, leading to increased vasodilation and decreased PVR [68].

Ambrisentan is a propanoic acid derivative with high selectivity for ET_A_ receptors [69]. This selectivity is a key characteristic distinguishing it from other ERAs. By specifically targeting ET_A_ receptors, ambrisentan inhibited the cellular proliferation and vasoconstriction driven by ET_A_ receptors on VSMCs, while maintaining the vasodilatory effects of ET_B_ receptors [70]. This selective action contributed to its efficacy in treating HAPH, and potentially minimizing unwanted side effects associated with non-selective ET receptor blockade [17,67].

Macitentan, a sulfamide, is a dual ERA similar to bosentan. It inhibited ET-1 from binding to both the ET_A_ and ET_B_ receptors [18]. However, its pharmacological profile differed from that of bosentan and ambrisentan due to its unique binding characteristics, particularly its slow receptor dissociation [71]. This slow dissociation allowed macitentan to more effectively block ET-1-induced signaling at the cellular level, potentially enhancing its therapeutic efficacy compared to other ERAs [72].

### 3.3 PI_2_ or prostacyclin receptor agonists

Selexipag, a selective prostacyclin IP receptor agonist, is renowned for its vasodilatory properties, along with its anti-proliferative, anti-inflammatory, and anti-thrombotic effects [73]. Selexipag induced vasodilation by agonizing the IP receptor [20]. Selexipag is chemically distinct from PGI_2_ and its analogs, as it is neither a PGI_2_ nor a PGI_2_ analog, but is still a highly selective oral prostacyclin IP receptor agonist. Notably, selexipag is metabolized by carboxylesterase 1, producing an active metabolite that is approximately 37 times more potent than the parent compound. Both selexipag and its metabolite exhibited high selectivity for the IP receptor over other prostanoid receptors. This selectivity ensured its efficient therapeutic action [73,74].

These pharmaceutical treatments have shown efficacy in treating HAPH; however, their long-term usage has been associated with side effects [21]. Therefore, integrating natural remedies and medicines can be a complementary approach to managing HAPH symptoms with potentially fewer side effects.

## 4 Natural medicinal herbal plants for managing HAPH

**Fig. 2:**
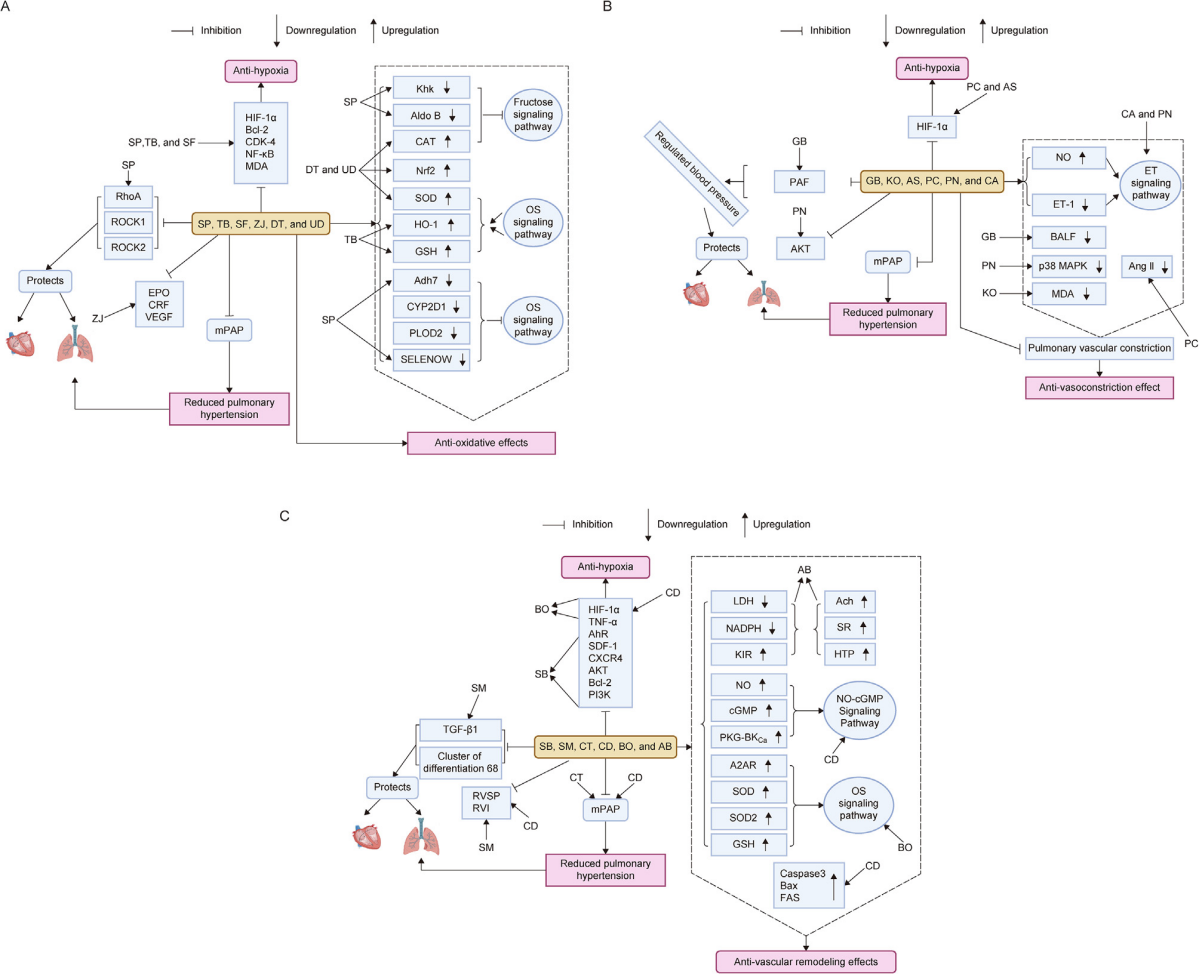
Effects of different medicinal plants on high-altitude pulmonary hypertension (HAPH) treatment: (A) anti-oxidative, (B) anti-vasoconstriction, and (C) anti-vascular remodeling effects. SP: *Salvia przewalskii*; TB: *Terminalia bellirica*; SF: *Sophora flavescens*; ROCK: Rho-associated protein kinase; ZJ: *Ziziphus jujuba*; EPO: erythropoieten; CRF: c-reactive protein factor; VEGF: vascular endothelial growth factor; HIF-1α: hypoxia inducible factor-1α; Bcl-2: B cell lymphoma 2; CDK-4: cyclin-dependent kinase 4; NF-κB: nuclear factor kappB; MDA: melanoaldehyde; DT: *Dracocephalum tanguticum*; UD: *Urtica dioica*; mPAP: mean pulmonary artery pressure; Khk: ketohexokinase; Aldo B: fructose-bisphosphate aldolase B; CAT: catalase; Nrf2: nuclear factor erythroide 2 related factor; SOD: superoxide dismutase; HO-1: hemeoxygenase-1; GSH: glutathione; Adh7: alcohol dehydrogenase 7; CYP2D1: cytochrome 2D1; PLOD2: procollagen-lysine 2-oxoglutarate 5-dioxygenase 2; SELENOW: selenoprotein W; OS: oxidative stress; GB: *Ginkgo biloba*; PAF: platelet activating factor; PN: *Panax notoginseng*; AKT: protein kinase B; PC: *Polygonum cuspidatum*; AS: *Allium sativum*; KO: *Kelussia odoratissima*; CA: *Conioselinum anthriscoides*; NO: nitic oxide; ET-1: endothelin 1; BALF: bronchoalveolar lavage fluid protein; p38 MAPK: p38 mitogen activated protein kinase; Ang II: angeotensinogen II; SM: *Salvia miltiorrhiza*; TGF-β1: tumor growth factor-beta 1; RVSP: right ventricular systolic pressure; RVI: right ventricular infarction; CD: *Cistanche deserticola*; BO: *Brassica oleracea*; SB: *Scutellaria baicalensis*; TNF-α: tumour necrosis factor-1alpha; AhR: aryl hydrocarbon receptor; SDF-1: stromal cell derived factor-1; CXCR4: C−X−C motif receptor 4; PI3K: phosphoinositide 3-kinase; CT: *Carthamus tinctorius*; AB: *Agaricus bitorquis*; LDH: lacatate dehydrogenase; NADPH: nicotinamide adenine dinucleatide; KIR: killer-Ig-like receptor; Ach: acetylcholine; SR: serotenin; HTP: hydroxytryptamine; cGMP: cyclic guanylate monophosphate (GMP); PKG-BK_C__a_: Ca^2+^-activated K^+^ channel; A2AR: adenosine 2A receptor; Bax: Bcl-2-associated X protein; FAS: fatty acid synthase. Figure created by BioRender.com.

**Fig. 3:**
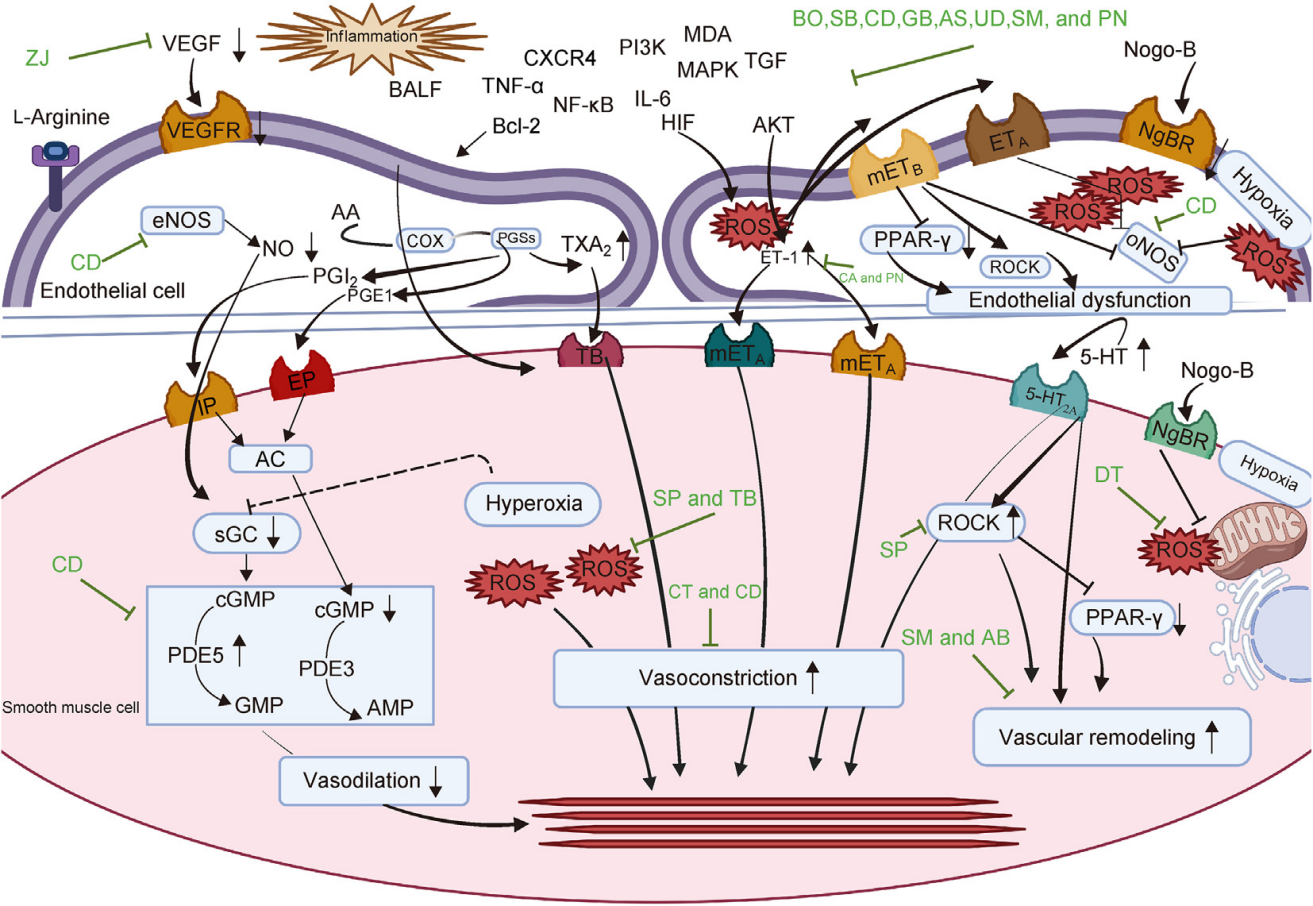
Mechanisms of action of medicinal plants targeting different biological pathways in high-altitude pulmonary hypertension (HAPH) by attenuating factors causing hypoxia, endothelial dysfunction, vasoconstriction, and vascular remodeling. ZJ: *Ziziphus jujuba*; VEGF: vascular endothelial growth factor; VEFGR: VEGF receptor; CD: *Cistanche deserticola*; eNOS: endothelial nitric oxide (NO) synthase; PGI_2_: prostacyclin; AA: arachidonic acid; COX: cyclooxygenase; PGS: prostaglandin synthase; PGE1: prostaglandin E1; TXA_2_: thromboxane A_2_; BALF: bronchoalveolar lavage fluid; TNF-α: tumour necrosis factor-alpha; CXCR4: C−X−C motif receptor 4; PI3K: phosphatidylinositol 3-kinase; MDA: melanoaldehyde; Bcl-2: B cell lymphoma 2; NF-κB: nuclear factor kappB; IL-6: interleukin 6; MAPK: mitogen activated protein kinase; TGF: tumor growth factor; BO: *Brassica oleracea*; SB: *Scutellaria baicalensis*; CD: *Cistanche deserticola*; GB: *Ginkgo biloba*; AS: *Allium sativum*; UD: *Urtica dioica*; SM: *Salvia miltiorrhiza*; PN: *Panax notoginseng*; HIF: hypoxia inducible factor; AKT: protein kinase B; ROS: reactive oxygen species; mET_B_: smooth muscle contractile endothelin (ET) receptor B; ET_A_: ET receptor A; Nogo-B: reticulon 4B; NgBR: Nogo-B receptor; CA: *Conioselinum anthriscoides*; PPAR-γ: peroxisome proliferator-activated receptor-γ; ROCK: Rho-kinase; eNOS: endothelial nitric oxide synthase; IP: inositol 1,4,5-trisphosphate receptors; EP: PGE1 receptor; TB: *Terminalia bellirica*; mET_A_: smooth muscle contractile ET receptor A; 5-HT_2A_: 5-serotonin (5-HT) receptor 2A; AC: adenylyl cyclase; sGC: soluble guanylate cyclase; cGMP: cyclic guanylate monophosphate (GMP); PDE5: phosphodiesterase-5; cAMP: cyclic adenosine monophosphate (AMP); SP: *Salvia przewalskii*; CT: *Carthamus tinctorius*; SM: *Salvia miltiorrhiza*; AB: *Agaricus bitorquis*; DT: *Dracocephalum tanguticum*. Figure created by BioRender.com.

Naturally occurring Chinese medicinal plants containing biologically active ingredients are increasingly recognized as natural medicines that can treat high-altitude diseases, including HAPH. These plants offered anti-oxidative, anti-vasoconstrictive, and anti-vascular remodeling effects (Fig. 2) by targeting specific molecular pathways implicated in the development of HAPH (Fig. 3). Detailed descriptions of these pharmaceutical effects induced by natural medicines are provided in this section.

### 4.1 Anti-oxidative effects

In the pathology of HAPH, the involvement of OS responses is widely acknowledged as pivotal. Natural medicinal plants exhibited potent antioxidant and anti-inflammatory properties by modulating expression of certain genes in specific molecular signaling pathways, making them valuable treatment candidates for HAPH pathology as shown in (Table S1 [[75], [76], [77], [78], [79], [80], [81]]). Most importantly, *Terminalia bellirica* (TB) (Combretaceae) [75], *Sophora flavescens* (SF) (Fabaceae) [76], *Urtica dioica* (UD) Linn*.* [77], *Ziziphus jujuba* (ZJ) Mill*.* (Rhamnaceae) [78], *Dracocephalum tanguticum* (DT) (Lamiaceae) [79], and *Salvia przewalskii* (SP) Maxim. (Lamiaceae) [80,81] exhibited potent antioxidant and anti-inflammatory properties.

Such as SF [76] exerted its preventive effects by upregulating the expression of SOD, heme oxygenase-1 (HO-1), and nuclear factor-E2-related factor 2 (Nrf2). SF exhibited anti-oxidative effects by attenuating levels of HIF-1α, nuclear factor kappB (NF-κB), and transforming growth factor-β1 (TGF-β1), thus reducing H_2_O_2_ levels in PASMCs by modulating OS signaling pathways, which reduced the production of ROS [76].

UD [77] also targeted OS signaling pathways, reducing ROS levels in PASMCs. ZJ [78], TB [75], and DT [79] were found to increase levels of SOD, B cell lymphoma-2 (Bcl-2)-associated X protein (Bax), plasma GSH peroxide (GSH-Px), Nrf2, and HO-1 [75,79,80], while they downregulated MDA and Bcl-2 in lung tissues and significantly inhibited ROS [75,78] by targeting the mitochondrial and OS signaling pathways.

Interestingly, SP was found to be an excellent source of bioactive ingredients, and its roots exhibited excellent therapeutic potential for managing the symptoms of HAPH [80,81]. This medicinal plant facilitated hypoxia adaptation by fructose metabolism inhibition via ketohexokinase and fructose-bisphosphate aldolase B (AldoB) downregulation [80]. In addition, SP was shown to reduce oxidoreductase activity by downregulating alcohol dehydrogenase 7 (Adh7), procollagen-lysine 2-oxoglutarate 5-dioxygenase 2 (PLOD2), cytochrome P2d1 (CYP2D1), selenoprotein W (SELENOW), nicotinamide adenine dinucleotide (NADH)-ubiquinone oxidoreductase chain 3 (ND3), and fatty acid-binding protein 1 (FABP1) by targeting OS signaling pathways [80]. Moreover, SP extracts were shown to downregulate HIF-1α, proliferating nuclear antigen, lactate dehydrogenase (LDH), Bcl-2, cyclin-dependent kinase 4 (CDK4), and cyclin D1 (CD1) through an anti-hypoxic mechanism in the mitochondrial pathway. The extracts also inhibited NF-κB and monocyte chemoattractant protein-1 (MCP-1), while modulating the Ras homolog family member A-Ras homolog (RhoA-Rho)-associated protein kinase signaling pathway, hence repairing the chronic hypoxia-induced lung injury [80,81]. Collectively, SP exhibited potent antioxidant and anti-hypoxic activity, making it a promising candidate for HAPH treatment.

### 4.2 Anti-vasoconstrictive effects

NO serves as a pivotal vasodilator, and its diminished availability along with increased levels of ET-1 and angiotensin II triggered pulmonary vasoconstriction [82]. HAPH pathology also relies on p38 MAPK pathway activation as an underlying mechanism in the progression of HAPH. Natural medicinal plants exhibited potent anti-vasoconstrictive effects by modulating expression of certain genes in specific molecular signaling pathways, making them valuable treatment candidates for HAPH pathology as shown in (Table S2 [[83], [84], [85], [86], [87], [88]]). For example, *Panax notoginseng* (PN) (Araliaceae) [83], *Polygonum cuspidatum* (PC) (Polygonaceae) [84], *Conioselinum anthriscoides* (CA) (Apiaceae) [85], *Ginkgo biloba* (GB) (Ginkgoaceae) [86], *Allium sativum* (AS) (Amaryllidaceae) [87], and *Kelussia odoratissima* Mozzaf (KO) (Apiaceae) [88] mitigated pulmonary vasoconstriction by specifically modulating certain pathways involved in HAPH and ultimately upregulated NO levels in the lungs by modulating the ET-1 signaling pathway.

CA has been found to alleviate ROS markers, ET-1 levels, and increase NO levels, hence significantly improving pulmonary vasodilation in pulmonary arteries by modulating the ET-1 pathway [85]. Although there are still few inconsistencies in the understanding of its mechanism of action on ET-1 inhibition through modulation of the ET-1 pathway, this medicinal plant is widely utilized to manage the HAPH pathology [85].

PN has been shown to attenuate p38 MAPK expression by targeting the p38 MAPK pathway, thereby increasing synthesis of NO and upregulating levels of NO in plasma and lungs, as evidenced by an *in vivo* study [83]. The mechanism of action of AS involved both endothelium-dependent and -independent mechanisms to positively influence pulmonary artery circulation, hence improving HAPH symptoms [87].

Interestingly, GB specifically antagonized platelet-activating factor (PAF) receptors to alleviate abnormal changes in the pulmonary systolic blood pressure. In addition, GB was shown to decrease levels of bronchoalveolar lavage fluid proteins, specifically TNF-α, TGF-β1, and interleukin-6 (IL-6) in alveolar cells, to exert its anti-inflammatory effects. PC regulated NO by attenuating levels of ET-1 and angiotensin II in the serum and lungs *in vivo* by targeting the ET-1 signaling pathway [84].

KO produced a significant rise in circulatory concentrations of NO, lowered the serum MDA levels, hematocrit, and heterophil/lymphocyte ratio, and attenuated right ventricular hypertrophy, hence demonstrating efficacy in combating HAPH with its vasodilation effects [88].

### 4.3 Anti-vascular remodeling effect

The thickening of pulmonary vessel walls eventually leads to pulmonary vascular remodeling during hypobaric hypoxia in HAPH pathology. Natural medicinal plants demonstrated high efficiencies for reducing PASMCs proliferation and reversing pulmonary vascular remodeling in HAPH pathology as shown in (Table S3 [[89], [90], [91], [92], [93], [94], [95], [96], [97], [98], [99], [100]]). Most importantly, *Salvia miltiorrhiza* (SM) (Lamiaceae) [[89], [90], [91]], *Scutellaria baicalensis* (SB) (Lamiaceae) [92], *Carthamus tinctorius* (CT) (Asteraceae) [93], *Brassica oleracea* (BO) (Brassicaceae) [94], *Agaricus bitorquis* (AB) (Agaricaceae) [95], *Cistanche deserticola* (CD) (Orobanchaceae) [[96], [97], [98]], *Rhodiola algida* (RA) (Crassulaceae) [99], and *Astragalus membranaceus* (AM) (Fabaceae) [100] reversed the pulmonary vascular remodeling in HAPH pathology.

SB [92] inhibited hypoxia-induced PASMC proliferation by lowering TGF-β1 levels and upregulating A2a receptors in the stromal cell-derived factor-1/chemokine C−X−C motif receptor 4 (SDF-1/CXCR4) signaling pathways. Selective binding of the A2a receptor with CXCR4, followed by interaction between CXCR4 and SDF-1, mediated the phosphoinositide 3-kinase/protein kinase B (PI3K/AKT) signaling pathways, ultimately inhibiting PASMC proliferation.

Interestingly, SM has been reported to downregulate p27 and S-phase kinase-associated protein 2 (Skp2) and attenuated AKT phosphorylation in the AKT signaling pathway, therefore lowering inflammation and inhibiting PASMC proliferation evidenced by *in vitro* and *in vivo* studies [91]. SM also downregulated TGF-β1, and suppressed HIF-1α expression [90], ultimately inhibiting hypoxia-induced gene expression and PASMC proliferation in pulmonary adventitial fibroblast cells [90]. SM has also been reported to cause significant reductions in right ventricular infarctions and right ventricular systolic pressure [90] in HAPH mice models by exerting its excellent anti-vascular remodeling effects to manage HAPH pathology.

AB has been found to significantly reduce LDH and NADPH oxidase levels by targeting cellular metabolic pathways and increasing acetylcholine, 5-hydroxytryptamine, killer-Ig-like receptor (KIR), and serotonin levels, thereby attenuating PASMCs proliferation [95]. CT also attenuated hypoxia-induced proliferation of PASMCs and pulmonary artery remodeling *in vivo*; however, there is still ambiguity regarding the specific mechanism of action of this medicinal plant, which warranted further research on the specific up- and downregulated genes in this mechanism [93].

CD relaxed pulmonary arterial rings through NO-cGMP pathway in a dose-dependent manner [98], caused opening of K^+^ channels, Ca^2+^-activated K^+^channel (BK_Ca_), and KIR [98]. In addition, a significant decrease in the hemoglobin, mPAP, right ventricular hypertrophy index, hematocrit level, mean wall thickness (%) of the pulmonary arteries [96], and Bcl-2 and HIF-1α levels were reported [97]. A significant increase in caspase-3, Bax, and Fas expression was also observed by the action of CD [98]; thus, CD regulated the pulmonary artery endothelium and confirmed its efficiency in remodeling pulmonary arteries in HAPH pathology.

RA has shown its anti-vascular remodeling effects by significantly reducing mPAP and right ventricular hypertrophy. In addition, it decreased the thickness of the pulmonary small arteries in a study, suggesting a reversal of vascular remodeling [99]. This effect was crucial as it helped alleviating the structural changes in the pulmonary arteries that increased vascular resistance. Moreover, RA was shown to modulate the expressions of several critical cell cycle regulators [99]. It also downregulated proliferating cell nuclear antigen (PCNA) expression, being involved in DNA synthesis and cell proliferation. The reduction in CDK4 and CD1 expressions further indicated suppression of the cell cycle, as these proteins played essential roles in driving cells from the G1 to the S phase. Conversely, the upregulation of p27Kip1, a CDK inhibitor, suggested enhanced inhibitory control over cellular proliferation. These molecular changes make it a promising candidate for mitigating pulmonary vascular remodeling and reducing the overall burden of PH [99].

AM was found to inhibit the T follicular helper (Tfh) cells differentiation as well as IL-21 synthesis. Conversely, it promoted the T follicular regulatory (Tfr) cells differentiation, TGF-β, and L-10 production. AM also influenced the regulation of Tfh and Tfr cell differentiation by inhibiting mammalian target of rapamycin (mTOR) phosphorylation in the mTOR signaling pathway. Furthermore, it attenuated PASMCs proliferation, migration, and adhesion *in vitro.* Meanwhile, under hypoxic conditions, AM significantly downregulated levels of RhoA and upregulated p27 levels in PASMCs. This suggested that AM may modulate Tfh and Tfr cell responses to suppress pulmonary vascular remodeling [100].

### 4.4 Combined effects

Combining natural medicines for alleviating the symptoms of HAPH requires a nuanced understanding of their combined effects. Initially, we examined the primary effects of these natural medicines, individually. However, some natural medicines exhibited secondary and tertiary effects, such as antioxidant, anti-vasoconstriction, and anti-vascular remodeling effects.

SP [80,81], SM [[89], [90], [91]], and CD [[96], [97], [98]] stand out regarding their secondary and tertiary effects, in addition to their primary effects. SP is particularly notable for its strong antioxidant activity [80]. It reduced OS by modulating key pathways associated with oxidative damage and by lowering ROS levels [80,81]. This primary antioxidant effect is crucial for repairing damage caused by hypoxia, and it may also indirectly influence vascular function and remodeling [81].

SM also demonstrated potent antioxidant activity by targeting inflammatory and OS markers [90,91]. Beyond its antioxidant effects, it played a significant role in vascular remodeling by inhibiting the proliferation of PASMCs and reducing right ventricular pressure [[89], [90], [91]]. While its direct anti-vasoconstriction effects are less clear, its primary benefits in antioxidant activity and vascular remodeling are well established [91].

CD exhibited vasodilatory properties by relaxing pulmonary arterial rings through the NO-cGMP pathway, which helped to alleviate vasoconstriction [96]. However, CD further contributed to vascular remodeling by reducing the pulmonary artery wall thickness and improving overall vascular structure [97,98] and showed anti-oxidative effects by downregulating OS biomarkers. Its comprehensive ability to alleviate OS, vasoconstriction, and vascular remodeling makes it a versatile option for combined therapy.

## *5* *In vitro* and *in vivo* model studies and their effects on activity and mechanisms of natural medicines

This section firstly provided a brief overview of various *in vitro* and *in vivo* studies conducted to assess the anti-oxidative [[75], [76], [77], [78], [79], [80], [81]], anti-vasoconstriction [[83], [84], [85], [86], [87], [88]], and anti-vascular remodeling [[89], [90], [91], [92], [93], [94], [95], [96], [97], [98], [99], [100]] effects of natural medicines for HAPH discussed in the previous section. Then, we explored how these model studies influenced the activity and mechanisms of natural medicines by considering the implications of different study designs on the interpretation of their efficacy and mechanisms of action.

### 5.1 *In vitro* and *in vivo* study models

Different *in vitro* and *in vivo* research model studies have been conducted to evaluate anti-oxidative effects in the context of HAPH, including studies examining SF and TB*,* both *in vitro* and *in vivo*. Specifically, SF study investigated male Sprague-Dawley (SD) rats divided into control, hypoxic, oxymatrine-treated, and hypoxia + oxymatrine groups (with dosages of 50 mg/kg/body weight), and analyzed PASMCs *in vitro* [76]. In addition, a TB study employed male SD rats with hypoxia-induced HAPH treated with saline (1 mL/100 g/day), sildenafil (30 mg/kg/day), or TTR extract (100, 200, and 400 mg/kg/day), and included *in vitro* analyses using PAECs and H_2_O_2_ [75]. Other studies were exclusive *in vivo* studies, such as a UD study testing dioica oil (0%, 0.5%, 1%, and 1.5%) on broilers that developed HAPH at 2100 m of elevation [77] and ZJ study, examined the effects of plant extract (6.25 mg/kg) on SD rats under HAPH conditions [78]. In addition, DT study involved male Wistar rats treated with various doses (100, 300, 500 mg/kg/body weight) of DT [79], and SP studies used male SD rats with hypoxia induction and SP supplementation (1 g/day or 500, 1000, and 2000 mg/kg/day) [80,81].

Studies for evaluating anti-vasoconstriction effects using plant extracts to address hypoxia have typically used *in vivo* models, such as CA study involved acute hypoxia in dogs treated with ligustrazine (80 mg/kg) [85]. Similarly, AS was tested in male Wistar rats under acute hypoxia that received allicin (100 mg/kg) [87]. GB was studied in rats exposed to high altitude and administered GB (200 mg/kg) [96]. In a chronic hypoxia study, PN was given to male Wistar rats for four week with PN doses of 30 mg/kg [88], while PC was tested in male SD rats under low-pressure and hypoxic conditions with various doses of polydatin (5, 10, 20 mg/kg) for three weeks [84]. KO was evaluated in broilers exposed to high altitude (2100 m) and supplemented with whole plant extract at varying concentrations over a 42-day period [88].

Studies on anti-vascular remodeling effects of various plant compounds have also involved both *in vitro* and *in vivo* models. SB was tested using baicalin in male SD rat PASMCs under normoxic and hypoxic conditions, with various treatment groups and knockdown models, using 40 μmol/L baicalin for 24 h [92]. SM was evaluated *in vitro* with PASMCs from hypobaric hypoxia-exposed SD rats and *in vivo* using 160 mg/kg Danshensu administered to hypoxia-exposed SD rats [89]. Another *in vivo* study with Danshensu used SD rat models treated with 80, 160, and 320 mg/kg during and after hypoxia exposure, which revealed different effects in preventive versus therapeutic applications [90]. Tanshinone IIA from SM was tested *in vitro* (0, 3, 10, 30, and 50 μg/mL in PASMCs) and *in vivo* (male SD rats) and impacted protein levels and phosphorylation at 10 mg/kg/day dosage [91]. CT with hydroxysafflower yellow A (HSYA) was evaluated *in vivo* with Wistar rats, demonstrating dose-dependent (25, 50, 75, and 100 mg/kg/body weight) effects on pulmonary arterial pressure [93]. Furthermore, BO with sulforaphane (SFN) was tested both *in vitro* (PASMCs cells) and *in vivo* (male BALB/c mice) studies, in which SFN improved biomarkers in BALB/c mice exposed to hypoxia at a dosage of 2 mg/kg [94]. Moreover, AB polysaccharides were tested *in vitro* on PASMCs at 200 μg/mL for 24 h under hypoxic conditions [95].

CD with echinacoside showed significant reductions in pulmonary artery parameters and improved remodeling in *in vivo* models (male SD rats given 3.75, 7.5, 15, 30, and 40 mg/kg dosage and male Wister rats given 30, 100, and 300 μmol/L dosage), with additional *in vitro* assessments revealing inhibition of PASMCs proliferation and modulation of apoptosis-related proteins [[96], [97], [98]]. In addition, RA bioactive fractions were tested *in vivo* on male SD rats (62.5, 125, and 250 mg/kg dosage) under hypoxic conditions, resulting in significant reductions in right ventricular hypertrophy and improved arterial remodeling [99]. Finally, AM with *Astragalus* IV was evaluated both *in vitro* (PASMCs) and *in vivo* (C57BL mice given 20, 40, and 80 mg/kg) models, which highlighted its impact on PASMCs proliferation and signaling pathways under hypoxia conditions [100].

### 5.2 Effects of model studies on activity and mechanisms of natural medicines

The variability in animal models used for studying HAPH significantly influenced the research outcomes and their applicability [[101], [102], [103]]. Different species, such as rats and dogs, exhibited unique physiological responses and metabolic processes that can affect how they react to natural medicines. For example, dogs may metabolize compounds differently than rats, leading to variations in efficacy and side effects. Furthermore, the choice of the hypoxia model, acute versus chronic, impacted the relevance of the findings [76]. Acute hypoxia models, which typically involved short-term exposure to relatively lower oxygen levels, might not fully represent the long-term adaptations and pathophysiological changes seen in chronic HAPH [76,85]. Conversely, chronic models that simulated prolonged exposure to relatively lower oxygen levels may not capture the immediate therapeutic effects of treatments that could be significant in acute scenarios [85].

Variations in treatment protocols, including dosage, duration, and administration routes, further complicated the interpretation of results [[90], [91], [92], [93]]. For example, dosing regimens and administration routes that worked effectively in one model may not have the same impact in another due to differences in absorption and metabolism. This highlighted the importance of using multiple models to validate the effectiveness and safety of natural medicines [104]. Therefore, by comparing results across different species and under different conditions, researchers can better understand the mechanisms of action and therapeutic potential of natural compounds, leading to more accurate predictions of their efficacy in humans.

## 6 Toxicity concerns of natural medicines for HAPH

While most natural medicines are generally safer or have relatively lower toxicity, their potential adverse effects should not be disregarded. Assessing the toxicity of natural medicines is challenging due to their complex composition and variability. For example, the adverse effects and mortality associated with SP are dose-dependent. In an animal study, the median lethal dose (LD_50_) of SP administered via gavage was found to be 2547 mg/kg, with adverse effects occurring at 1981 mg/kg. Despite these findings, there remains a substantial safety margin for using SP as a natural medicine [105]. In comparison, the LD_50_ for bosentan in rats is around 1400 mg/kg when administered orally at 62.5 mg twice daily for four weeks, indicating higher toxicity [106] than that of SP. The notable side effect of bosentan is hepatotoxicity, necessitating regular liver function tests.

Meanwhile, no acute or subacute toxicity has been found with SM [107], SB [108], or CD [109]. However, SF exhibited mild toxicity at a dose of 256.74 mg/kg in mice when administered intravenously [110]. In comparison, the LD_50_ of ambrisentan in mice is about 2180 mg/kg when administered orally [69], with potential adverse effects that included elevated liver enzymes and peripheral edema, indicating a higher safety margin for SF. Acute neurotoxic, cytotoxic, and cardiotoxic effects have been observed with a 450 mg/kg dose of PN [111], leading to liver, heart, and brain damage. In comparison, the LD_50_ for macitentan in mice is approximately 135 mg/kg when administered orally, indicating significantly higher toxicity than that of PN. However, the LD_50_ of selexipag is 2000 mg/kg when administered orally [112], and has side effects including headache, diarrhea, and jaw pain.

GB exhibited fewer side effects [113] compared with sildenafil citrate [114], which has an LD_50_ of 1900 mg/kg when administered orally, with potential side effects that included headache, flushing, and dyspepsia. However, adverse cardiac events and serious bleeding have been associated with GB use [113]. While toxicity and potential cancer-related consequences have been observed in rodents, such occurrences have not been reported in humans and remain controversial.

Hence, it is crucial to implement standardized methodologies for toxicity assessments across various natural medicines, ensuring consistent and comparable results. Long-term studies should be prioritized to observe chronic effects, particularly for medicines with mild acute toxicity, providing a clearer understanding of their safety over prolonged use. Moreover, conducting well-designed human clinical trials is essential to validate findings from animal studies and assess real-world safety, particularly to identify potential drug interactions. Importantly, investigating the underlying mechanisms of toxicity can help to identify why specific doses or compounds caused adverse effects, guiding safer formulations. Furthermore, including diverse populations in these studies is important, as genetic, dietary, and lifestyle differences can significantly influence the effects of natural medicines.

## 7 Combined use of natural medicine in the treatment of HAPH

The combined use of natural medicines for treating HAPH may pose several critical considerations due to their potential interactions and cumulative side effects. For example, SP and SM, both known for their vasodilatory properties, can have an additive impact on blood pressure [115,116]. Their combined effect may benefit patients by reducing PH but also raised the risk of hypotension. Therefore, careful monitoring of blood pressure is essential for adjusting dosages appropriately and preventing adverse outcomes associated with excessively reduced blood pressure.

SM and SB both exhibited significant antioxidant and anti-inflammatory properties [89,90,92]. When used together, they provided enhanced cardiovascular benefits by reducing OS and inflammation, which are crucial for managing HAPH [92]. However, there is a potential risk of additive hypotensive effects, which necessitated monitoring for signs of low blood pressure to ensure that the benefits of these herbs outweigh any potential risks.

The combination of CD and SF was shown to provide a synergistic anti-inflammatory effect, which could be advantageous for treating HAPH by reducing pulmonary inflammation [75,98]. Nonetheless, caution is needed due to the mild toxicity associated with SF [110]. Regular safety evaluations and close monitoring are important to prevent adverse reactions and manage any potential toxicity. SF and PN both improved blood circulation due to their vasodilatory effects [76,83]. While this helped to alleviate symptoms of HAPH, there is also a risk of excessive vasodilation leading to hypotension.

PN has been linked to neurotoxic, cytotoxic, and cardiotoxic effects at high doses. Hence, careful dose adjustments and vigilant monitoring are required to manage potential toxicity and ensure safe use [117]. Moreover, combining PN with GB may enhance blood circulation [84], which is beneficial for HAPH pathology. However, the blood-thinning effect of GB increased the risk of bleeding [118]. This risk is particularly significant when combined with other herbal medicines that affected blood circulation [119]. Therefore, it is crucial to closely monitor for bleeding complications and managing dosages carefully to mitigate these risks. The tendency of GB to increase the bleeding risk can be exacerbated when used in combination with other herbal medicines that have anticoagulant effects. This interaction highlighted the importance of careful consideration of herbal combinations, particularly in patients who are already at risk for bleeding or who are on anticoagulant medications [118].

In summary, understanding these interactions when combining natural medicines involved a comprehensive approach integrating traditional knowledge with modern scientific methods. This included assessing the pharmacological interactions of the herbs, conducting rigorous clinical trials for evaluating their safety and efficacy, and implementing regular safety monitoring to detect and manage adverse effects in a timely manner. By employing this informed strategy, healthcare providers can enhance the effectiveness of natural medicines in treating HAPH, maximizing therapeutic benefits and reducing potential risks.

## 8 Exploring alternative candidate natural medicines for HAPH

*Zanthoxylum armatum* (*Z. armatum*) (Rutaceae) boasts a rich reservoir of bioactive compounds, such as polyphenols and flavonoids, which play a pivotal role in neutralizing harmful free radicals and maintaining cellular health [120]. Across various traditional medicinal practices, *Z. armatum* has been used to address a spectrum of health ailments. Notably, studies have documented its efficacy in managing AMS, along with its therapeutic potential for treating hypertension endocrine and metabolic disorders [121,122]. Interestingly, research studies have indicated that the active component of *Z. armatum*, tambulin, acted as an endothelium-independent vasodilator by exerting its influence on VSMCs within the coronary artery [121]. Hence, tambulin holded promise for addressing conditions characterized by dysregulated blood flow in HAI. While existing findings underscored the potential therapeutic benefits of *Z. armatum* in mitigating AMS, further exploration is warranted, particularly for HAPH treatment, considering its pulmonary vasodilation, anti-hypoxic, and anti-oxidative effects.

*Xenophyllum poposum* (*X. poposum*) (Phil.) V.A. Funk (Asteraceae) has a long medicinal history. Its aerial parts served as a potent remedy for an array of health concerns, including hypertension, gastrointestinal disorders, and rheumatism [123]. Emerging research has highlighted the remarkable antioxidant and cardioprotective properties inherent in the extract of *X. poposum*. This botanical marvel demonstrated negative inotropic effects as well as vasodilation effects by modulating vascular endothelial cells and regulating intracellular calcium concentrations, ultimately culminating in improved heart function [123]. Despite its local prominence, the global literature on the efficacy of *X. poposum* in treating HAIs remained scant. The limited availability of comprehensive studies underscored the pressing need for more rigorous investigation into its therapeutic potential, particularly for treating HAPH. Only through the accumulation of high-quality evidence can we unravel the intricacies of the mechanisms of action of *X. poposum* and ascertain its precise role in addressing altitude-related health challenges.

*Pleurospermum lindleyanum* (*P*. *lindleyanum*) (Apiaceae) is renowned for its medicinal properties and harbors a plethora of active constituents, including flavonoids, coumarins, and monoterpenes [124]. Notably, *P*. *lindleyanum* has demonstrated potent antioxidant properties, as evidenced by its ability to elevate plasma NO levels and enhance SOD activity *in vivo* in hypertensive mice [125]. However, while the existing research provided promising insights, critical questions regarding its efficacy, mechanism of action, and safety in the treatment of HAPH remain unanswered. Further exploration is warranted to elucidate the precise role of *P. lindleyanum* in mitigating altitude-related health challenges and to pave the way for its safer and effective integration into clinical practice.

Therefore, considering their proven abilities to treat ailments closely related to hypertension, these plants are identified as promising candidates for further *in vitro* and *in vivo* research, particularly in the context of HAPH. Hence, further research specifically focusing on these promising natural candidates is highly encouraged for treating HAPH.

## 9 Conclusion and future perspectives

HAPH remains a significant concern for people at high altitudes, with increasing travel and residence in such areas. Current pharmaceutical treatments manage symptoms but come with side effects and limitations for long-term use, prompting interest in alternative therapies. Recent research into natural medicines, like Chinese herbal remedies and plant-based treatments, shows promise as safer options but requires further validation through rigorous studies to confirm their efficacy and safety. Future research should focus on understanding the pharmacological mechanisms and bioavailability of these natural remedies, assessing their long-term efficacy, and exploring new treatment options. Integrating traditional and modern approaches could enhance treatment strategies, combining conventional drugs with natural therapies to maximize benefits and minimize adverse effects. Collaborative research across various scientific disciplines will be crucial in developing more effective and personalized treatments, ultimately improving patient outcomes and quality of life for those affected by HAPH.

## CRediT authorship contribution statement

**Zahra Batool:** Data curation, Validation, Visualization, Writing – original draft, Writing – review & editing. **Mohammad Amjad Kamal:** Formal analysis, Investigation. **Bairong Shen:** Funding acquisition, Resources, Supervision, Writing – review & editing.

## Declaration of competing interest

The authors declare that there are no conflicts of interest.
