# Supplementary material for: Advanced treatment strategies for high-altitude pulmonary hypertension employing natural medicines: A review
Source: J Pharm Anal. 2024 Oct 25;15(3):101129. doi: 10.1016/j.jpha.2024.101129 (PMC11953983; doi:10.1016/j.jpha.2024.101129)
Supplement: Multimedia component 1 [file mmc1.docx]

**Table S1.** Summary of studies denoting anti-oxidative effects by medicinal plants evidenced by *in vitro* and *in vivo* molecular studies

| Plant Binomial ( Family) | Bio-active compounds | Study Type | Dosage | Study Design | Pharmaceutical effect | References |
| --- | --- | --- | --- | --- | --- | --- |
| *Terminalia bellirica*  (Combretaceae) | *Terminalia bellirica* (Gaertn.) Roxb. (TTR extract) | *In vitro* and *In vivo* | 100, 200 and 400 mg/kg/day (*In Vivo*) and  20, 40, or 80 µg/mL (*In Vitro*) | Male SD rats induced with HAPH divided into 5 groups: (1) a model group receiving normal saline (1 mL/100 g/day), (2) a sildenafil group receiving sildenafil tablets (30 mg/kg/day), (3) TTR-H group (400 mg/kg/day), (4) TTR-M group (200 mg/kg/day), and (5) TTR-L group (100 mg/kg/day). A 15 days treatment in the high altitude, while controls remained in the plain environment.  For *in vitro* experiment, PAECs divided into 5 groups: (1) normal control group, (2) model group incubated for 1 h with 800 µM H2O2, and (3) TTR at concentrations of 20 µg/mL, (4) 40 µg/mL, and (5) 80 µg/mL, followed by incubation for 1 h with 800 µM H2O2 24 h after the intervention. | Significant alleviation of mPAP, or slowed pulmonary arterial remodeling,  Elevated SOD and GSH-Px, Nrf2, Bax, and HO-1. While decreased Bcl-2 and MDA in lung tissues *in vivo*. Moreover, dose dependent inhibition of H_2_O_2_-induced PAECs apoptosis and ROS production, downregulated Bcl-2 and upregulated Bax, Nrf2, and HO-1  expression in PAECs *in vitro.* | [75] |
| *Saphora flavescens* (Fabaceae) | Oxymatrine | *In vitro* and *in vivo* | 50 mg/kg/bw | Adult male Sprague-Dawley rats divided into 4 groups: (1) control, (2) hypoxic group—rats housed in a chamber containing 10% oxygen for continuous exposure to hypobaric hypoxia, (3) oxymatrine-treated group—rats receiving a daily dose of 50 mg/kg of oxymatrine alone, and (4) hypoxia + oxymatrine group—rats exposed to hypoxia and simultaneously treated with oxymatrine for 28 days. Subsequently, pulmonary artery smooth muscle cells (PASMCs) isolated from pulmonary rings for *in vitro* study. | Significant inhibition of proliferation of PASMCs, HIF-1α and NF-κB and ROS levels with attenuation of pulmonary vasoconstriction.  Significant increase in the levels of SOD, GSH, HO-1 and Nrf2. | [76] |
| *Urtica dioica* (Urticaceae) | 96.7 % dioica oil | *In vivo* | 0, 0.5%, 1%, 1.5% oil in mL | Broilers were subjected to HAPH induction at an altitude of 2100 meters for six weeks, with continuous administration of dioica at concentrations of 0%, 0.5%, 1%, and 1.5%. | Significant increase in SOD and CAT levels in liver and lungs of broilers fed with dioica with dose dependent effect. Significant rise in NO and MDA concentrations. Moreover, high does (1 % and 1.5 %) attenuated right ventricular hypertrophy . | [77] |
| *Ziziphus jujuba* (Rhamnaceae) | Fruit extract | *In vivo* | 6.25 mg/kg | Male and female SD rats were induced with HAPH using a hypobaric chamber. Following induction, one group received treatment with 6.25 mg/kg of fruit for three weeks, while the controls received no treatment. | Significant decrease in EPO, CRP, VEGF levels in serum with inhibition of inflammatory reaction in the treatment group. | [78] |
| *Dracocephalum tanguticum* (Lamiaceae) | Whole plant | *In vivo* | 100,300,500 mg/kg/bw | Male Wistar rats were induced with HAPH using a hypoxic chamber, followed by supplementation with different doses of dracocephalum for two weeks. The groups were as follows: (1) control group with no dosage, (2) supplementation with 100 mg/kg of dracocephalum, (3) supplementation with 300 mg/kg of dracocephalum, and (4) supplementation with 500 mg/kg of dracocephalum for the two-week period. | Significant reduction in MDA, mPAP and RVI in rats lung tissue. However, SOD, GSH-Px levels were significantly increased with dose dependent effect. | [79] |
| *Salvia przewalskii Maxim* (SPM) (Lamiaceae) | Dried SPM | *In vivo* | 1.0 g/kg/day | Male SD rats divided in 3 groups: (1) normal group, (2) hypoxia group exposed to an altitude of 4260 meters, and (3) hypoxia + SPM group exposed to an altitude of 4260 meters with supplementation of 1.0 g/kg/day of SPM for 4 weeks. | Significant decrease in mPAP, and downredulation of HIF-1α, Adh7, Cyp2d1, plod2, selenow, ND3, FABp1, Khk and Aldob. | [80] |
| *Salvia przewalskii Maxim* (SPM) (Lamiaceae) | Dried SPM | *In vivo* | 500, 1000, 2000 mg/kg | Male SD rats subjected to hypoxia induction at an altitude of 4260 meters, followed by administration of doses of SPM before and after hypoxia induction in the treatment groups. The groups were categorized as follows: (1) control group receiving no dose, (2) treatment group receiving 500 mg/kg of SPM, (3) treatment group receiving 1000 mg/kg of SPM, and (4) treatment group receiving 2000 mg/kg of SPM for a duration of three week. | Significant decease in RhoA, mPAP, ROCK1, and ROCK2 levels in dose dependent manner. | [81] |

PAECs: pulmonary artery endothelial cells; mPAP: mean pulmonary arterial pressure; GSH-Px: plasma glutathione peroxidae; SOD: superoxide dismutase; MDA: malonodialdehyde; Bax: bcl-2-associated x protein; HAPH: high altitude pulmonary hypertension; Bcl-2: b cell lymphoma-2; Nrf2: nuclear factor erythroide 2 related factor; HO-1: heme oxygenase-1; H_2_O_2_: hydrogen peroxide; ROS: reactive oxygen species; PASMCs: pulmonary arterial smooth muscle cells; HIF-1α: hypoxia inducible factor-1; NF-κB: nuelear factor κB; GSH: glutathione; CAT: catalase; NO: nitric oxide; EPO: erythropoieten; CRP: c-reactive Pprotein, VEGF: vascular endothelial growth factor ; RVI: right ventricular infarction; Adh7: alcohol dehydrogenase 7; Cyp2d1: cytochrome P2 d1; plod2: procollagen-lysine 2-oxoglutarate 5-dioxygenase 2; Selenow: selenoprotein W; ND3: nadh-ubiquinone oxidoreductase chain 3; FABp1: fatty ccid-binding protein; Khk: ketohexokinase; Aldob: fructose-bisphosphate aldolase B; RhoA: ras homologue gene family member A; ROCK: rho-associated protein kinase

**Table S2.** Summary of studies denoting anti-vasoconstrictive effects by medicinal plants evidenced by *in vitro* and *in vivo* molecular studies

| Plant Binomial ( Family) | Bio-active compounds | Study Type | Dosage | Study Design | Pharmaceutical effect | References |
| --- | --- | --- | --- | --- | --- | --- |
| *Panax notoginseng* (Araliaceae) | Panax notoginseng saponins (PNS) | *In vivo* | 30 mg/kg | Male Wistar rats subjected to a four-week hypoxia protocol followed by daily intraperitoneal injections of PNS (at a dose of 30 mg/kg) before being placed into the hypoxic chamber. | Significant decrease in P38 MAPK in the lungs with increased NO levels in lungs as well as plasma. | [83] |
| *Polygonum cuspidatum* (Polygonaceae) | Polydatin | *In vivo* | 5, 10, 20 mg/kg | Male SD rats divided in groups (1) Normal oxygen group and normal control group; (2) In the hypobaric group and hypoxia group, 0.5 mL of normal saline was intraperitoneally injected under low pressure and hypoxia conditions (3) In the positive control group, rats were given 1.7 mg/kg of silaenafil intragastrically 10 min before hypoxia and hypoxia (4) In the low-dose polydaddin group, rats injected with 5 mg/kg polydaddin intraperitoneally, and hypoxia was induced. (5) In the medium-dose polydaddin group, rats injected with 10 mg/kg polydadin intraperitoneally every 10 days of hypotension and hypoxia; (6) In the high-dose polydadin group, rats intraperitoneally injected with 20 mg/kg polydadin 10 min for low pressure and hypoxia. Rats are subjected to low-pressure and hypoxic conditions for 8 h per day by exposing the rats to an auto-regulated low-atmospheric pressure (50 kPa) and hypoxic (10% oxygen) chamber. This study was carried out for 3 consecutive week. | Lowered hypoxic pulmonary hypertension, reversed remodeling, as well as regulated the levels of NO, ET, AngII in dose dependent manner. However, the effects of polydatain was mitigated by PKC forced activation and signalingvia THX activation. | [84] |
| *Conioselinum anthriscoides* (Apiaceae) | Ligustrazine | *In vivo* | 80 mg/kg | Acute pulmonary hypoxia was induced in dogs using a hypoxic chamber, followed by intravenous injections of ligustrazine at a dosage of 80 mg/kg for a period of three weeks in the treatment group. | Inhibition of OS markers and ET-1 levels as well as pulmonary vasoconstriction | [85] |
| *Ginkgo biloba* (Ginkoaceae) | Ginkgolide B | *In vivo* | 200 mg/kg/bw | Male Wistar rats were induced with HAPH by exposing them to high altitude (380 mmHg pressure) for 24 hours. Then provided with Ginkgolide B in their drinking water at a dosage of 200 mg/kg/body weight for two days both before and after exposure to high altitude. | Significant decrease in BALF protein and PAF. | [86] |
| *Allium Sativum* (Amaryllidaceae) | Allicin | *In vivo* | 100 mg/kg/bw | Alveolar hypoxia was induced in male Wistar rats, followed by oral gavage administration of Allicin for five consecutive days in the treatment group. | Significant inhibition of pulmonary vasoconstriction and hypoxic pulmonary hypertension | [ 87] |
| *Kelussia odoratissima Mozzaf* (Apiaceae) | Whole plant extract | *In vivo* | 0%, 0.25%, 0.5%, and 0.75% | 208-day-old male broilers (Ross 308) were induced with HAPH by exposing them to an altitude of 2100 meters. They were then randomly assigned to four treatment groups, each receiving different levels of Kelussia odoratissima Mozzaf supplementation (0%, 0.25%, 0.5%, and 0.75%) over a 42-day trial period. Additionally, three additional diets were prepared by substituting 0.25%, 0.50%, and 0.75% of Kelussia odoratissima Mozzaf for wheat bran in the control diet. | Significant higher circulatory concentrations of NO and lower serum MDA hematocrit and heterophil to lymphocyte ratio compared with birds fed with control diet. Moreover, 0.5 % and 0.75 % treatments attenuated right ventricular hypertrophy. | [88] |

P38 MAPK: mitogen activated protein kinase p38; NO: nitric oxide; Ang II: angeotensinogen II; ET: endothelin; THX: thymeleatoxin; OS: oxidative stress; HAPH: high altitude pulmonary hypertension; BALF: [bronchoalveolar lavage fluid](https://www.sciencedirect.com/topics/medicine-and-dentistry/bronchoalveolar-lavage-fluid) protein; PAF: antagonising platelet activating factor; MDA: malondialdehyde

**Table S3.** Summary of studies denoting anti-vascular-remodeling effects by medicinal plants evidenced by *in vitro* and *in vivo* molecular studies

| **Plant Binomial ( Family)** | **Bio-active compounds** | **Study Type** | **Dosage** | **Study Design** | **Pharmaceutical effect** | **References** |
| --- | --- | --- | --- | --- | --- | --- |
| *Salvia miltiorrhiza* (Lamiaceae) | Danshensu | *In vitro* and *in vivo* | 160 mg/kg/bw | Male SD rats were exposed to hypobaric hypoxia for a duration of four weeks. Following this exposure, they were administered 160 mg/kg of Danshensu *in vivo*. Subsequently, the proliferation of rat PASMCs and TGF-β pathway investigated *in vitro*. | Significant inhibition of hypoxia- or TGF-β-induced proliferation of PASMCs.  Downregulation of hypoxia-induced expression and secretion of transforming growth factor in primary pulmonary adventitial fibroblasts and NR8383 cell line, inhibited the hypoxia or TGF-β-induced phosphorylation in rat PASMCs. | [89] |
| *Salvia miltiorrhiza* (Lamiaceae) | Danshensu | *In vivo* | 80, 160, 320 mg/kg/bw | Male SD rats divided into (1) Normoxic (N), (2) hypoxic (H) groups. Group 1 remained untreated, while group 2 placed in hypobaric chamber and depressurized to 380 mmHg (reduced oxygen concentration about 10%), 8 hours a day. Group 2 received 80 mg/kg, 160 mg/kg danshensu and 320 mg/kg danshensu treatment, separately.  For the preventive study, danshensu was intraperitoneally injected daily into Group 2 rats at doses of 80 mg/kg, 160 mg/kg, and 320 mg/kg, beginning when the rats underwent exposure to hypoxia and continuing for 4 weeks.  For the therapeutic study, danshensu was intraperitoneally injected at the same doses for 2 weeks after rats had been exposed to hypoxia in Group 2. | Significant reduction in RVSP and RVI and TGF-β in preventive group while, no significant improvement observed in the therapeutic group. | [90] |
| *Salvia miltiorrhiza* (Lamiaceae) | Tanshinone IIA | *In vitro* and i*n vivo* | 0, 3, 10, 30 and 50 µg/mL (*In vitro*), 10 mg/kg/day (I*n vivo*) | Male SD rats sacrificed to obtain PASMCs, which were then cultured in either normoxic (21%) or hypoxic (3%) conditions for *in vitro* study. Prior to culture, the PASMCs were treated with varying concentrations of Tanshinone IIA (0, 3, 10, 30, and 50 µg/mL) for 24 hours.  Male SD rats divided in (1) (a) normoxia (control) and (b) hypoxia (reduced 10% O_2_) (control), (2) normoxia plus tanshinone IIA (10 mg/kg/day), (3) hypoxia plus tanshinone IIA (10 mg/kg/day) groups intraperitoneally injected 10 mg/kg/day tanshinone IIA every day for 3 week for *in* *vivo* study. | Significant down-regulation of Skp2, p27 protein levels in PASMCs, and attenuation of AKT phosphorylation *in vitro* and *In vivo*. | [91] |
| *Scutellaria baicalensis* (Lamiaceae) | baicalin | *In vitro* and *in vivo* | 40 μmol/L | Male SD rats PASMCs cultured under normoxia/hypoxia in 9 groups (1) normoxia, (2) hypoxia, (3) hypoxia + AMD3100 (a CXCR4 antagonist), (4) hypoxia + baicalin, (5) hypoxia + negative virus, (6) normoxia + A2aR knockdown, (7) hypoxia + A2aR knockdown, (8) hypoxia + CGS21680 (an A2aR agonist), and (9) hypoxia + A2aR knockdown + baicalin. Lentiviral transfection established the A2aR knockdown model in PASMCs. Cells were incubated under hypoxic conditions for 24 h. | Significant alleviation of hemodynamic changes and pulmonary arterial remodeling, SDF-1, CXCR4, P13K and AKT phosphoryl, HIF-1α and AhR in baicalin treated hypoxia group. However, A2aR expression was increased. | [92] |
| *Carthamus tinctorius* (Asteraceae) | Hydroxysafflower Yellow A (HSYA) | *In vivo* | 25, 50, 75, 100 mg/kg/BW | Male Wistar rats induced with HAPH by subjecting them to a hypoxic chamber for 24 hours per day for up to 9 days. Some rats received treatment with doses of HSYA (25,50,75 and 100 mg/kg/BW), while others did not receive any treatment. Subsequently, the pulmonary arterial pressure was measured to assess the effectiveness of the treatment. | Significant reduction in PASMCs proliferation as well as pulmonary artery remodeling on all treatments with dose dependent effect | [93] |
| ***Brassica oleracea*** (Brassicaceae) | Sulforaphane (SFN) | *In vitro* and i*n vivo* | 2 mg/kg | Male BALB/c mice assigned to 4 groups: (1) normoxia group, (2) normoxia+SFN 2mg/kg group, (3) hypoxia group, and (4) hypoxia+SFN 2 mg/kg group. Hypoxic mice were housed in a hypobaric chamber with 10% oxygen, while normoxic mice were housed in normobaric conditions with 21% oxygen.  SFN was administered daily by gavage for a duration of 5 weeks, with a pre-administration period of 1 week. Mice in the normoxia and hypoxia groups were given a 1% DMSO solution in PBS.  Subsequently, PASMCs were isolated from the mice tissues for *in vitro* testing. | Reduction in CD68 positive cells in lung sections,  Significant decrease in TNF-*α* and IL-6 levels ,  Significant elevation of SOD, SOD2 expression, GSH levels, Moreover,  *in vitro* inhibition of PASMCs proliferation as well as promoted PASMCs apoptosis in SFN treated hypoxia induced group. | [94] |
| *Agaricus bitorquis* (Agaricaceae) | Water soluble Polysaccharides | *In vitro* | 200 ug/mL | PASMCs were subjected to *in vitro* hypoxia induction, followed by treatment with 200 μg/mL of polysaccharides. | Significant reduction in the levels of LDH and NADPH oxidase levels and increased Ach, 5HTP, SR and KIR6.2 levels. | [95] |
| *Cistanche deserticola* (Orobanchaceae) | Echinacoside (ECH) | *In vivo* | 3.75, 7.5, 15, 30 and 40 mg/kg | [Male SD rats](https://www.sciencedirect.com/topics/pharmacology-toxicology-and-pharmaceutical-science/sprague-dawley-rat) divided in ((1) normoxia group (control); (2) chronic [hypoxia](https://www.sciencedirect.com/topics/pharmacology-toxicology-and-pharmaceutical-science/hypoxia) group (HPH rat model); (3) ECH (3.75 mg/kg) group; (4) ECH (7.5 mg/kg) group; (5) ECH (15 mg/kg) group; (6) ECH (30 mg/kg) group; (7) ECH (40 mg/kg) group.) housed in a hypobaric [hypoxia](https://www.sciencedirect.com/topics/pharmacology-toxicology-and-pharmaceutical-science/hypoxia) chamber (4500 m) for 28 days except control group. Different concentrations of ECH were administered by [intraperitoneal injection](https://www.sciencedirect.com/topics/pharmacology-toxicology-and-pharmaceutical-science/intraperitoneal-injection) from the 1st to the 28th day to animals in 3 and 7 group. | Significant and dose dependent reduction in mPAP, hematocrit, right ventricular hypertrophy index and mean wall thickness (%) of pulmonary arteries. Echinacoside regulated pulmonary artery endothelium and smooth muscle layer function and improved the remodeling of pulmonary artery. | [96] |
| *Cistanche deserticola* (Orobanchaceae) | Echinacoside (ECH) | *In vitro* and *in vivo* | 30, 100, 300 μmol/L | Male Wister rats pulmonary artrial rings and their PASMCs were treated with (30, 100, 300 μmol/L) concentrations of ECH for 24 hours (1) control with no treatment (2) 30 μmol/L (3)100 μmol/L (4) 300 μmol/L. | ECH relaxed pulmonary artrial rings in dose dependent manner by NO-cGMP pathway and opening of K+ channels (BKCa and KIR). Under extracellular Ca2+-free conditions, the maximum contraction  was reduced to 24.54%±2.97% and 10.60%±2.07% in rings treated with 100 and 300 μmol/L of ECH. Moreover, dose dependent proliferation inhibition in PASMCs was also obtained. | [97] |
| *Cistanche deserticola* (Orobanchaceae) | Echinacoside (ECH) | *In vitro* and *in vivo* | 0.35 mM, 0.4 mM | PASMCs from 6-8 week old male Wister rats were incubated under (1) normoxia (nor),(2) hypoxia (hyp), (3) hypoxia + 0.35 mM ECH (hyp + ECH0.35), or (4) hypoxia + 0.4 mM ECH (hyp + ECH0.4) for 24 h. | Significant inhibition of hypoxia induced rats PASMCs proliferation.  Significant increase in caspase 3, Bax and Fas expression. However, decrease in expression of Bcl-2 and HIF-1α. | [98] |
| *Rhodiola algida* (Crassulaceae) | *Bioactive fraction from R. algida* (ACRT) | *In vivo* | 62.5, 125 and 250 mg/kg | SPF male SD rats were randomly assigned to one of five experimental groups: 1: Control group: Rats were maintained under normal conditions without any treatment. 2: Hypoxia group: Rats were placed in a hypobaric chamber, with the pressure and oxygen content adjusted to simulate the conditions at an altitude of 4500 meters for a duration of 4 weeks. 3: Hypoxia + ACRTs (low dose) group: Rats were subjected to the same hypoxic conditions as the hypoxia group and received ACRTs at a dose of 62.5 mg/kg/day for 4 weeks. 4: Hypoxia + ACRTs (medium dose) group: Rats were exposed to the hypoxic environment and treated with ACRTs at a dose of 125 mg/kg/day for 4 weeks. 5: Hypoxia + ACRTs (high dose) group: Rats experienced the hypoxic conditions and were administered ACRTs at a dose of 250 mg/kg/day for 4 weeks. Each group was monitored throughout the study to evaluate the effects of ACRTs on rats exposed to high-altitude hypoxia. | Significantly reduced mPAP and right ventricular hypertrophy. It also decreased the thickness of the pulmonary small arteries and down-regulated the expression of PCNA, CDK4 and CD1. However, caused up-regulation of p27Kip1. | [99] |
| *Astragalus membranaceus* (Fabaceae) | *Astragalus IV* | *In vitro* and *In vivo* | 20,40 and 80 mg/kg | Sixty C57BL mice were randomly divided into 6 groups (n=10): control group (NaCl), model group, model + sildenafil group (SN,100 mg/kg, positive control), model + low-dose ASIV group (ASIV-L, 20 mg/kg ASIV), model + medium-dose ASIV group (ASIV-M, 40 mg/kg ASIV) and model + high-dose ASIV group (ASIV-H, 80 mg/kg ASIV. Mice exposed to ASIV housed in hypoxia (~380 mmHg, 2-21 days). Control mice were exposed to ambient air pressure (normoxia, N; ~630 mmHg). Mice in the ASIV-L group, ASIV-M group and ASIV-H group intraperitoneally injected with ASIV once a day for 21 days. Animals in the SN group were intraperitoneally injected with sildenafil citrate once a day for 21 days.After 21 days, mice were sacrifised and their PASMCs were removed for further *in vitro* study. | Significantly inhibited the differentiation of Tfh cells and the production of IL-21 and promoted the differentiation of Tfr cells and the production of TGF-β and L-10. Inhibited mTOR phosphorylation in the mTOR signaling pathway. Furthermore, it inhibited the proliferation, migration, and adhesion of PASMCs *in vitro.* Meanwhile, significantly down-regulated RhoA protein levels while up-regulating p27 kip1 protein levels in PASMCs. | [100] |

PASMCs: pulmonary artery smooth muscle cells; TGF-β; tumor growth factor-β ; RVI: right ventricular infarction; Skp2: s-phase kinase-associated protein 2; AKT: serine threonine kinase; SDF-1: stromal cell derived factor; CXCR4: cxc chemokine receptor 4; P13K: phosphoinositide 3 kinase; HIF-1α: hypoxia inducible factor-1α; AhR: aryl hydrocarbon receptor; A2aR: adenosine 2a receptor; DMSO: dimethylsulfoxide; TNF*:* tumor necrosis factor; IL-6: interleukine-6; SOD: superoxide dismutase; GSH: glutathione; LDH: lactata dehydrogenase; NADPH: nicotinamide-adenine dinucleotide phosphate; Ach: acetylcholine; 5HTP: 5-hydroxytryptamine; SR: serotinine; KIR: killer- Ig-like receptor; NO: nitric oxide; cGMP: cyclic guanosine monophosphate ; BKCa: c**a2**+-activated k**+** channel; Bax:bcl-2-associated x protein; Fas: fatty acid synthase; Bcl-2 : B cell lymphoma-2; HIF-1α: hypoxia inducible factor-1α; SPF: specific pathogen-free; SD: sprague-dawley; mPAP: mean pulmonary arterial pressure; PCNA: proliferating cell nuclear antigen, CDK4: cyclin-dependent kinase 4 ; CD1: cyclin d1 ; p27Kip1: kinase inhibitor protein 1 ; NaCl: sodium chloride; Tfh: T follicular helper; I L: interluekin; mTOR: mammalian target of rapamycin ; RhoA: rho-associated protein kinase
